# Possibilities for Relapsing Fever Reemergence

**DOI:** 10.3201/eid1203.050899

**Published:** 2006-03

**Authors:** Sally J. Cutler

**Affiliations:** * Veterinary Laboratories Agency, Surrey, United Kingdom

**Keywords:** perspective, relapsing fever, Borrelia recurrentis, Borrelia duttonii, tickborne infections, louseborne infections, spirochetes, travel medicine

## Abstract

Relapsing fever *Borrelia* infections have attracted little attention in recent years; however, where endemic, these infections still result in considerable illness and death. Despite the marked antimicrobial drug susceptibility of these organisms, therapy is often delayed through lack of clinical suspicion. With increasing travel, infections may be imported, through exotic relapsing fever infection or through resurgence of infected disease vectors. Although louseborne relapsing fever is now geographically limited, it was once of global importance. The possibility for reemergence was recently highlighted by the probable reemergence of louseborne relapsing fever in homeless persons from France. Host limitations enforced through louseborne transmission are less applicable for the tickborne forms of relapsing fever. Although the latter have reduced potential for epidemic spread, they have the ability to infect diverse hosts, thus establishing reservoirs of infection and presenting greater challenges for their control.

## Abstract 2

Increasing globalization may pave the way for reemergence of relapsing fever.

Since the 1980s, the number of *Borrelia* species associated with relapsing fever has doubled. This situation is in part due to improved diagnostics and molecular techniques that have enabled sequenced-based characterization of these spirochetes. Seventeen species are attributed to the relapsing fever group of spirochetes; others have been described but await further characterization and isolation before species can be designated. Infection is vectorborne, primarily by *Ornithodoros* ticks, which led to the descriptive name of tickborne relapsing fever. However, 1 form of the disease developed epidemic potential by adapting to louse transmission and thus became known as louseborne relapsing fever.

Clinically, these spirochetes all produce an undulating febrile disease in humans, with signs and symptoms often indistinguishable from those of malaria. Diagnosis in most disease-endemic areas relies on demonstrating the spirochete in Giemsa-stained blood films; however, more discriminating methods are available that can be used in suitably equipped facilities.

Whether we are likely to see the reemergence of this disease is difficult to predict because we do not understand relapsing fever borreliae and their complex host interactions. Indeed, to use the words of Bryceson et al., "Little is understood of where it lurks between epidemics and of how it suddenly springs up after silent intervals of several years" (*1*). The same authors describe the louseborne disease as the "most epidemic of the epidemic diseases." Certainly, increasing population movements and travel to disease-endemic areas are likely to introduce relapsing fever to areas where it is not been common. Here, the danger lies in not considering this diagnosis or mislabeling the disease as Lyme borreliosis because of the likely cross-reactive serologic results. Although the louseborne epidemic form of the disease was once distributed globally, it is now localized to a few countries. The recent documentation of probable cases in homeless populations in France raises the possibility of reintroduction of this disease in countries where it was believed to have been eliminated.

## Discovery of the Disease

Compatible clinical disease descriptions have been documented since the time of Hippocrates; however, the term relapsing fever was first used by David Craigie to describe an outbreak of the disease in Edinburgh in 1843 (*1*). The spirochetal cause for louseborne relapsing fever was first demonstrated by Otto Obermeier during an outbreak in Berlin (1867–1868). His inability to reproduce the disease in animal models (and indeed in himself) delayed the publication of these findings until 1873 (*1*). The causative agent of the African tick variety of relapsing fever was discovered by Ross and Milne in 1904 (*2*). This finding was also made independently by Dutton and Todd, who demonstrated transmission by using infected *Ornithodoros moubata* ticks and a monkey model (*3*). The publication of their findings in 1905 resulted from the fact that Dutton became infected with this spirochete. The role of the human body louse in the transmission of relapsing fever was reported by MacKie in 1907 (*1*).

## Historical Epidemics and Endemic Disease Foci

During the first half of the 20th century, relapsing fever was a disease of major worldwide importance; it caused epidemics affecting ≈50 million and was associated with death rates of 10% to 40% (*1*). During the 1930s, approximately one third of the population in Africa was devastated by an epidemic attributed to relapsing fever. Since 1967, the epidemic form of louseborne relapsing fever has been largely confined to areas of extreme poverty in East Africa (*4*) and the Peruvian Andes; most cases occur in Ethiopia. A recent outbreak in neighboring Sudan is estimated to have affected 20,000 members of the Dinka tribe in 1998 and 1999; the death rate was 10%–14%. Despite the reappearance elsewhere in the world of other louseborne diseases, such as epidemic typhus in Burundi and trench fever in vagrant populations, little evidence of reemergence of louseborne relapsing fever exists. Furthermore, molecular analysis of lice collected from around the world, including France, Peru, Russia, and the African countries of Burundi, Congo and Zimbabwe, did not produce evidence of infection with the louseborne relapsing fever agent (*5*).

**Figure 1:**
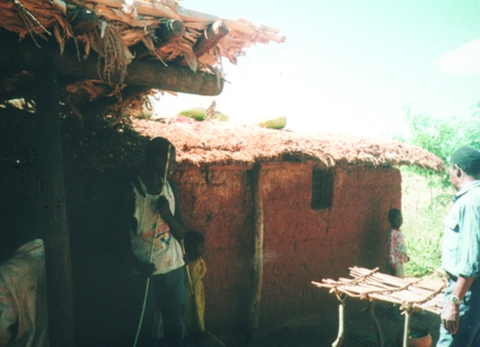
*Ornithodoros moubata* ticks that frequent traditional homes.

Tickborne relapsing fevers may be endemic or sporadic. They still cause major health problems in Africa; in areas such as central Tanzania, this disease is a substantial cause of child deaths (*4**,**6*). Although present in some European countries, Central Asia, the Middle East, and the Americas, tickborne relapsing fever tends to be rarer (*7**–**10*). It is often associated with camping out in rural locations in close proximity to animal reservoirs of the spirochete and their associated *Ornithodoros* tick vectors (Figure 1) (*7**–**9*).

## Relapsing Fever Today

This disease still has persistent foci of infection, where control can be a major healthcare problem. Relapsing fever can be acquired by travelers and brought back to regions where the disease is not epidemic (*10**,**11*) after eco-challenges or in association with military training or activities such as camping or caving, provided susceptible hosts and natural disease ecologic cycles coincide (*8**,**9*). Although many would argue that this limited disease impact is not a threat to public health, the lack of consideration of relapsing fever as a potential cause of clinical findings is a cause for concern.

## Tickborne Relapsing Fevers

*Borrelia duttonii*, the cause of tickborne relapsing fever, is endemic to several countries in East Africa, such as Tanzania. No accurate data on the number of cases in Tanzania exist because this infection is not reportable in the Ministry of Health's Health Management Information System. Estimates of the incidence are likely to be grossly underestimated because many cases are diagnosed as malarial treatment failures. Localized studies have shown that the annual incidence is 384/1,000 in children <1 year of age and 163/1,000 in children <5 years of age (*4*), while other researchers have reported an incidence of 59/1,000 (*12*). *B. duttonii* infection primarily occurs in children and pregnant women, and it is associated with fetal loss and neonatal deaths. A perinatal death ratio of 436/1,000 has been reported from disease-endemic regions of Tanzania (*4*).

**Figure 2:**
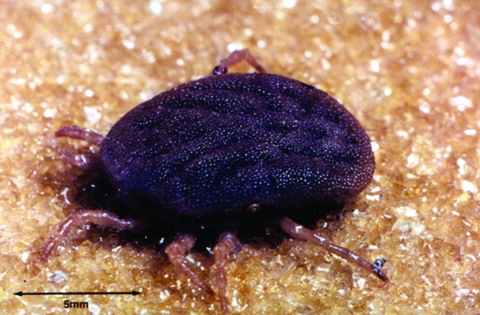
Traditional Tembe dwelling in Tanzania.

**Figure 3:**
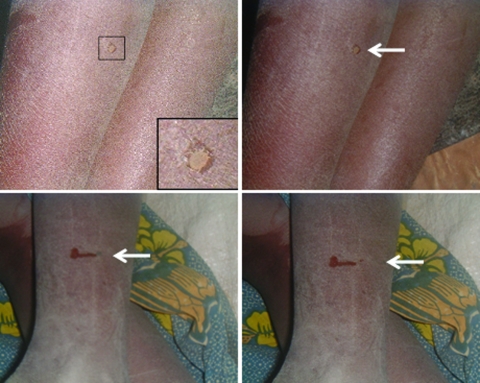
*Ornithodoros moubata* ticks feeding on sleeping children.

The vector is the soft tick, genus *Ornithodoros*; the species complex *Ornithodoros moubata* is prevalent in sub-Saharan Africa. These ticks live in traditional housing and mainly feed nocturnally (Figures 2 and 3). The disease is transmitted either by saliva during tick feeding or in coxal fluid excreted during feeding. The tick feeds for a short time only (usually less than half an hour), then returns to the earth floor or walls of the house. Humans are believed to be the only natural reservoir for *B. duttonii*, unlike the situation for *B. crocidurae* in West Africa, where rodents are reservoirs. (Up to 18% of rodents from Senegal are infected with this spirochete [13]). Human infection with *B. crocidurae* appears to be more prevalent than previously described, however (*14*). This increase could be attributed to improved diagnostic capabilities and sample processing (*15*), but it is largely due to molecular techniques that can facilitate not only detection but also subsequent identification through sequencing the target gene (*16**,**17*).

Reports of tickborne relapsing fever elsewhere appear to be more sporadic or involve limited clusters of infection, which typically are associated with events that bring susceptible human hosts into close proximity with the tick vectors and their usual wildlife reservoirs (*8**,**9*). The species most frequently incriminated in such cases include *B. turicatae* and *B. hermsii*. Isolated reports of *B. persica* infection transmitted by its *O. tholozani* vector have also appeared in the Middle East (*9*), and *B. hispanica* transmission has been reported in Spain and North Africa (*10*).

## Louseborne Relapsing Fevers

Many now believe that louseborne relapsing fever can be assigned to the history books; however, considerable disease-endemic foci of infection remain in areas of Ethiopia, which spill into neighboring countries such as Sudan (*18*). This global reduction of louseborne relapsing fever has largely resulted from the demise of the human clothing louse through improved human living conditions. A cause for concern is the increasing preference for washing clothes at low temperatures, which could permit louse survival. Ideally, clothes should be washed in water >60°C to kill lice. These lice are unable to survive away from their human hosts for more than a matter of hours and consequently tend to persist in areas of extreme poverty or in association with major population upheavals and turmoil, such as wars or environmental disasters. The increase of clothing lice among less privileged persons in industrialized countries is a growing concern. Blood samples collected from homeless persons in France from 2000 to 2003 showed serologic reactivity against *B. recurrentis*, which suggests a small disease outbreak and serves as a reminder against complacency against controlling or eliminating this disease (*19*).

## Potential for Introduction or Reintroduction into Industrialized Countries

The ability of these spirochetes to remain associated with their arthropod vectors throughout the lifespan of the vector is well established (*20*). However, most show limited capacity for transovarial transmission (*21*) since these spirochetes are typically transmitted through soft ticks or clothing lice, both of which only feed for a short duration (<30 minutes) before sequestering in cracks and crevices or seams of clothing.

Neither soft ticks nor lice can "travel" from their particular ecologic niches. However, finding *Borrelia* spp. that greatly resemble relapsing fever borreliae with natural transmission through hard tick (ixodid) vectors means that these ticks may play a role in transmission. Ixodid ticks have been found in association with seafaring birds and songbirds, which raises the possibility of migration-associated transmission. Furthermore, the finding of novel *Borrelia* spp. in Africa that strongly resemble New World relapsing fever species suggests greater globalization of these spirochetes (*22**–**24*). As yet, the possible clinical importance of these spirochetes, for which the name "*Candidatus* Borrelia mvumii" has been proposed, remains unresolved (*16**,**22**,**23*). Studies are particularly hampered by the current lack of cultivatable strains.

Studies of rodents in California have shown a new potential borrelial species, tentatively named "*Borrelia davisii*," which appears distinct from both the Lyme-associated and relapsing fever groups of spirochetes. Despite the finding of this spirochete in blood samples collected from rodents, *Ixodes scapularis* ticks collected from the same geographic region were uniformly negative, which indicates that an alternative vector may exist for this spirochete. The clinical importance, if any, remains to be ascertained.

Lice are restrained by their exquisite host specificities. Human clothing lice have been adapted to feeding on a rabbit host (*20*); however, they still show strong preference for their human hosts. Whether other species of lice, or indeed other arthropod vectors, could transmit louseborne spirochetes is an open question. More recently, excretion of *B. recurrentis* in louse feces has been demonstrated (*20*). Louse feces, in turn, could become aerosolized through desiccation and dispersal, potentially increasing possibilities for spread of this infection; however, this route of transmission currently is anecdotal.

The possibility that the agents of louseborne epidemic relapsing fever (*B. recurrentis*) and of East African tickborne relapsing fever (*B. duttonii*) may indeed be the same, or highly related clones of a common ancestor, has been raised (*24*). Phylogenetic comparisons have demonstrated the homology between these spirochetes and resemblance with the West African *B. crocidurae* species (*16**,**17**,**24*). More recently, analysis of an intragenic spacer typing method for these spirochetes, although showing clear differences between *B. crocidurae*, was unable to discriminate between *B. recurrentis* and *B. duttonii* (*24*). If indeed these strains are homogeneous, the potential transmission of the louseborne *B. recurrentis* by tick vectors must be considered.

Travel patterns have changed in recent years, particularly in more affluent nations; remote rural areas are particularly popular. Thus, imported cases associated with tourists returning to their home countries are likely (*10*). Vacation-associated infection with *B. hermsii* and *B. turicatae* has been reported from the Americas (*8*), while travel-associated cases of *B. crocidurae* and *B. hispanica* infection have been reported in Europe (*10**,**15*). Marked differences in the incidence of disease have been noted between civilian populations, who are becoming increasingly urbanized, and military populations, who have greater potential contact with rural environments during training exercises (*9*). Subsequent febrile illness is likely to be diagnosed as malaria; however, microscopic examination of Giemsa-stained blood films should show the spirochetal cause of such cases. Of concern is the ability of spirochetes to persist within the blood of patients long after fever resolves (*10*). Several mechanisms facilitate this persistence, including antigenic variation, binding of factor H, and rosetting of erythrocytes (*25**–**27*). Furthermore, increased movements of animals, their produce, and even fomites make alternative vehicles for transportation of these diseases likely, either through undiagnosed infection or transport of the vectors themselves. This was likely the route for introduction of West Nile virus into the United States.

## Reservoirs of Infection and Vector Control

### Natural Reservoirs

The role of wildlife as natural reservoirs for *Borrelia* spp. (particularly rodents, warthogs, or other mammalian hosts) is well established; most relapsing fevers rarely affect humans (*13**,**28*). In contrast, *B. duttonii* and *B. recurrentis* have no identified natural hosts other than humans. For these 2 spirochetes, whether the vector is also the reservoir (with the human's role being amplification of infected vectors) or if humans can be considered as the reservoir for infection is debatable. Recent work by Kisinza et al. in the Dodoma region of Tanzania found 5% of febrile children had positive blood slides for *Borrelia* spp (*22*). Others studying nonfebrile persons have reported that 3% of them are spirochetemic by blood film examination. Furthermore, our own population studies on incidence in randomly chosen villagers in 4 Tanzanian villages showed that 11% were positive for borrelial DNA by using polymerase chain reaction. Whether this level of infection is sufficient to sustain the disease is not known.

### Livestock

The role of livestock as hosts is not well established; however, a recent case report described equine abortion associated with a spirochete resembling *B. parkeri* and *B. turicatae* (*29*). This case also indicates that infection may be associated with livestock transportation. Whether other livestock or companion animals are susceptible to infection remains to be established.

### Vectors

Many diseases now considered to be emerging or reemerging threats are vectorborne. Factors contributing to this phenomenon include changes to natural habitats, climatic change, and different levels of vector control strategies.

The stringent host specificity of the louse vector is the probable reason for the current demise of louseborne relapsing fever. In contrast, tick vectors are less host-specific; thus, spirochetes can be transmitted to more diverse hosts. Short-term vector control methods can be instigated, such as through use of acaricides. Although these methods have met with some success, we also need more affordable, yet sustainable, means of intervention that are likely to break the natural transmission cycles or reduce contact between reservoir, vector, and susceptible hosts (*6*). Improvements in housing design or institution of physical barriers such as bed nets could reduce vectors' access to susceptible hosts. Although these measures could reduce disease under certain circumstances, they offer little protection for populations at high risk, such as the homeless in industrialized countries.

## Host-Pathogen Relationships

Spirochetes must be maintained within the circulatory system of the host, thus enabling transmission to uninfected co-feeding or subsequently feeding arthropod vectors. Host blood persistence appears to be the result of many interactions between the host and spirochete. The ability of these spirochetes to undergo antigenic variation is well documented (*25*). Briefly, successive waves of spirochetemia characteristically seen in relapsing fever are associated with different antigenic variants, which result from rearrangement of silent variable membrane protein genes into an expression locus. This repertoire may be further extended through the multiplicity of mosaiclike variable membrane protein genes and pseudogenes possessed by some members of this group. These genes may have a role in extending antigenic diversity akin to the combinational gene conversion described in *Anaplasma marginale* (*30*).

This description does not address the question of whether spirochetes associated with clinical cases differ from those that do not appear to cause overt clinical signs. Immunity is a factor, highlighted by the high prevalence of disease in young children and pregnant women. However, for humans to serve as reservoirs for this disease, the spirochete must persist in sufficient numbers in a site where they will be acquired by new arthropod vectors. An intriguing hypothesis is that particular antigenic variants of the spirochete may be associated with life in an arthropod vector (usually ticks, except for the related spirochete, *B. recurrentis*), while others are suited to persistence in their human host (disease reservoir); additional types are associated with patients with overt clinical signs mediated through a multiplicity of host-microbe interactions. Persistence may be further facilitated through the neurotrophic tendency of these spirochetes. This hypothesis is further strengthened by observed pathologic differences associated with isogenic variants of the same isolate of *B. turicatae* investigated in vivo (*31*).

Clinical manifestations and patient management fall largely outside the scope of this review. However, clinical findings are reported elsewhere (*1*), in particular, details of management of neurologic, respiratory, and cardiac complications of these diseases.

Both innate and acquired immune responses are needed to control infection. To overcome innate immune mechanisms, these spirochetes bind the complement inhibitor factor H to their surface, thus avoiding deposition of the membrane attack complex and subsequent lysis (26; Meri et al., unpub. data). Clearance of spirochetes is primarily mediated through production of specific bactericidal immunoglobulin M antibodies (*32*); complement is not required (*33*). This specific immunoglobulin is primarily directed against the outer membrane lipoproteins of these spirochetes. T cells do not appear to have a major role in the resolution of spirochetemia (*32*).

Another mechanism that may enhance persistence of these spirochetes within the bloodstream is the ability of some relapsing fever spirochetes to become coated with or to rosette blood erythrocytes. This process enables prolonged persistence through a masking or steric hindrance effect, which prevents interaction with the host immune cells (*27**,**34*). This ability to bind erythrocytes might be associated with pathologic observations, including vascular tissue damage, hemorrhages, and inflammation (*27*); however, this hypothesis would not explain similar pathologic observations in nonrosetting phylogenetically related borreliae.

## Future Prospects

Traditionally, diagnosis is based on demonstration of spirochetes in blood films taken during the acute febrile period. More recently, an enzyme-linked immunosorbent assay that used the *GlpQ* gene product was demonstrated to be a useful diagnostic aid (*35*). Furthermore, this antigen is specific for the relapsing fever group borreliae, thus distinguishing these from cases of Lyme borreliosis (*35*).

Characterization of relapsing fever *Borrelia* spp. can now be facilitated through molecular approaches that allow more accurate investigation of which species are prevalent in different epidemiologic foci. Previously, such characterization relied on identification of disease vectors and compatibility with established geographic patterns. Furthermore, these techniques will enable characterization of newly discovered spirochetes (*36*) and identification of their reservoirs of infection. For example, spirochetes that were described in the guts of termites have now been allocated to a separate genus (*37*). Additional spirochetes have been identified in soil; however, their taxonomic status and clinical importance remain to be established (*38*). Other spirochetes have been discovered in various tick species, some showing rapid in vitro growth (*39*) and others producing no cultivable isolates to date (*22*).

Genome sequencing is under way for several relapsing fever spirochetes. This information will enable a thorough comparative analysis of these spirochetes and likely yield insights into vector competence and pathogenicity.
